# Body Surface Area Predicts Plasma Oxaliplatin and Pharmacokinetic Advantage in Hyperthermic Intraoperative Intraperitoneal Chemotherapy

**DOI:** 10.1245/s10434-012-2790-8

**Published:** 2013-03-02

**Authors:** Joshua C. Leinwand, Gleneara E. Bates, John D. Allendorf, John A. Chabot, Sharyn N. Lewin, Robert N. Taub

**Affiliations:** 1 Division of Medical Oncology, Department of Medicine, Columbia University Medical Center, New York, NY USA; 2 Department of Surgery, Columbia University Medical Center, New York, NY USA; 3 Division of Gynecologic Oncology, Department of Obstetrics and Gynecology, Columbia University Medical Center, New York, NY USA

## Abstract

**Background:**

Hyperthermic intraoperative intraperitoneal chemotherapy (HIPEC) is used to treat peritoneal surface-spreading malignancies to maximize local drug concentrations while minimizing systemic effects. The pharmacokinetic advantage of HIPEC is defined as the intraperitoneal to intravascular ratio of drug concentrations. We hypothesized that body surface area (BSA) would correlate with the pharmacokinetic advantage of HIPEC. Because oxaliplatin is administered in 5 % dextrose, we hypothesized that BSA would correlate with glycemia.

**Methods:**

We collected blood and peritoneal perfusate samples from ten patients undergoing HIPEC with a BSA-based dose of 250 mg/m^2^ oxaliplatin, and measured drug concentrations by inductively coupled plasma mass spectrophotometry. We monitored blood glucose for 24 h postoperatively. Areas under concentration-time curves (AUC) were calculated by trapezoidal rule. Pharmacokinetic advantage was calculated by (AUC[peritoneal fluid]/AUC[plasma]). We used linear regression to test for statistical significance.

**Results:**

Higher BSA was associated with lower plasma oxaliplatin AUC (*p* = 0.0075) and with a greater pharmacokinetic advantage (*p* = 0.0198) over the 60-minute duration of HIPEC. No statistically significant relationships were found between BSA and blood glucose AUC or peak blood glucose levels.

**Conclusions:**

Higher BSA is correlated with lower plasma drug levels and greater pharmacokinetic advantage in HIPEC, likely because of increased circulating blood volume with inadequate time for equilibration. Plasma glucose levels after oxaliplatin HIPEC were not clearly related to BSA.

The use of hyperthermic intraoperative intraperitoneal chemotherapy (HIPEC) with oxaliplatin has been reported for peritoneal carcinomatosis from colorectal cancer, ovarian carcinoma, pseudomyxoma peritonei, and malignant peritoneal mesothelioma.^1^ A goal of HIPEC is to maximize local drug exposure to areas of tumor while limiting systemic drug exposure; the ratio of local to systemic drug concentrations is known as the pharmacokinetic advantage.^2^ Because of oxaliplatin’s instability in chloride-containing solutions, 5 % dextrose is a frequently used carrier fluid during HIPEC.^3^ As in intravenous chemotherapy, the dose of oxaliplatin during HIPEC is usually calculated based on body surface area (BSA).^1^ Some institutions dilute the drug in a standard volume of carrier fluid, some calculate carrier fluid volume based on BSA, and some titrate carrier fluid volume to achieve a desired flow rate during HIPEC.^1^,^4^,^5^ As a result, there is variability between patients in the concentration of oxaliplatin in the perfusate. Likewise, the duration of chemoperfusion has not been standardized; perfusion times range from 30 min to 2 h.^1^,^6^

In a previous study, the absorption of oxaliplatin during HIPEC was associated with body mass index (BMI).^4^ The goals of the current study were to test whether BSA or BMI predict local or systemic exposure to oxaliplatin, or glycemia, during and after HIPEC.

## Patients and Methods

On an institutional review board approved protocol and with informed consent, peritoneal fluid and blood samples were collected during closed-technique HIPEC in ten patients with pseudomyxoma peritonei (*n* = 5), malignant peritoneal mesothelioma (*n* = 4), or peritoneal carcinomatosis from colon cancer (*n* = 1). Patients received a BSA-based oxaliplatin dose of 250 mg/m^2^ in 5 % dextrose carrier fluid titrated to achieve a flow rate of 1 L/min over a 60-minute chemoperfusion. Samples were analyzed using inductively coupled plasma mass spectrophotometry. Blood glucose was analyzed for 24 h following HIPEC. For the 60-minute duration of HIPEC (samples at 10, 30, and 60 min) and 24-hour blood glucose levels, area under concentration-time curve (AUC) was calculated by trapezoidal rule, BSA determined by DuBois and Dubois formula, and pharmacokinetic advantage by (AUC[peritoneal fluid]/AUC[plasma]).^7^ Peritoneal cancer index (PCI) and completeness of cytoreduction (CC) scores were determined for all patients.^8^,^9^ Linear regression was performed using SAS 9.2.

## Results

Baseline characteristics of all patients, including PCI and CC scores are listed in Table 1. One patient had a PCI score of 0, as he had previously undergone cytoreduction without any gross disease recurrence, and HIPEC only was performed, without any resection.

**Table 1:** Baseline characteristics of all patients, extent of disease and surgical treatment

ID	Diagnosis	Age (years)	Sex	PCI	CC	Extent of peritonectomy	Resections	Prior resections
1	Peritoneal mesothelioma	79	M	3	0	Right diaphragm	None	Omentectomy
2	Pseudomyxoma peritonei	65	F	5	0	Pelvis	Omentectomy, TAH-BSO	None
3	Pseudomyxoma peritonei	57	F	4	0	Bilateral paracolic gutters	Omentectomy, TAH-BSO	None
4	Pseudomyxoma peritonei	48	F	2	0	None	Right hemicolectomy	TAH-BSO
5	Colon cancer	61	F	12	0	None	Right hemicolectomy, TAH-BSO	None
6	Peritoneal mesothelioma	63	F	2	0	None	Omentectomy	None
7	Peritoneal mesothelioma	65	M	15	0	Right paracolic gutter, left diaphragm	Omentectomy, splenectomy	None
8	Pseudomyxoma peritonei	25	F	6	0	Bilateral diaphragms, bilateral paracolic gutters	None	Omentectomy, appendectomy, right salpingo-oophorectomy
9	Pseudomyxoma peritonei	63	F	15	0	Bilateral diaphragms	Omentectomy, splenectomy, appendectomy, TAH-BSO	None
10	Peritoneal mesothelioma	68	M	0	0	None	None	Omentectomy

*F* female, *M* male, *PCI* peritoneal cancer index score, *CC* completeness of cytoreduction score, *TAH*-*BSO* total abdominal hysterectomy–bilateral salpingo-oophorectomy

**Fig. 1:**
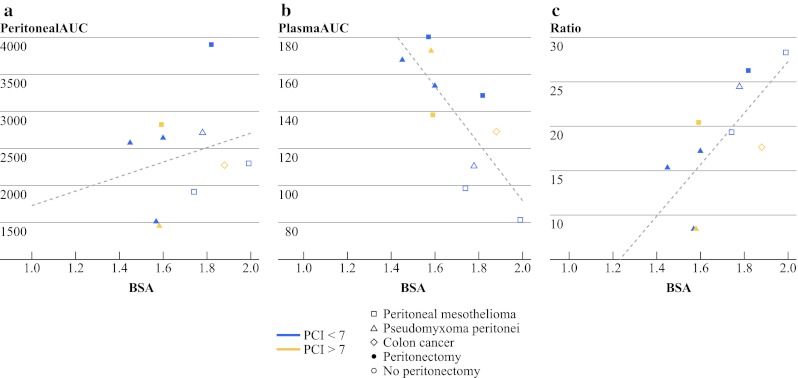
Linear regression plots of body surface area versus oxaliplatin pharmacokinetic parameters. BSA (m^2^) versus **a** peritoneal fluid AUC (mg min/L) (*p* = 0.5273), **b** plasma AUC (mg min/L) (*p* = 0.0075), and **c** pharmacokinetic advantage (*p* = 0.0198)

We examined perfusate volume, BSA and BMI as independent variables; of these, only perfusate volume and BSA were significantly correlated. Overall pharmacokinetic parameters and Pearson correlation coefficients with perfusate volume, BSA and BMI and as independent variables are listed in Table 2. Higher perfusate volume was associated with lower plasma oxaliplatin AUC (β = −30.7 mg min/L^2^, *p* = 0.0170). Higher BSA was associated with lower plasma oxaliplatin AUC (β = −153.2 mg/m^2^·min/L, *p* = 0.0075), and with a greater pharmacokinetic advantage (β = 28.7/m^2^, *p* = 0.0198) over the 60-minute duration of HIPEC. There were no statistically significant relationships between perfusate volume and peritoneal fluid oxaliplatin AUC or pharmacokinetic advantage, or between BSA and peritoneal fluid oxaliplatin AUC, or between BMI and any of the pharmacokinetic parameters. The relationships between BSA and oxaliplatin pharmacokinetic parameters are depicted in Fig. 1. There did not appear to be differences in pharmacokinetics based on diagnosis, extent of peritonectomy or between patients with greater or lesser burdens of disease, as measured by PCI with a cutoff of seven.

**Table 2:** Pharmacokinetic parameters and Pearson correlation coefficients

	Mean (SD)	Correlation with perfusate volume (*p* value)	Correlation with BSA (*p* value)	Correlation with BMI (*p* value)
Perfusate volume (L)	2.7 (0.8)	–	0.788 (0.0068)	0.130 (0.7205)
BSA (m^2^)	1.70 (0.17)	–	–	0.095 (0.7935)
BMI (kg/m^2^)	25.8 (4.6)	–	–	–
Plasma AUC (mg min/L)	138.1 (33.1)	−0.728 (0.0170)	−0.782 (0.0075)	−0.054 (0.8820)
Peritoneal fluid AUC (mg min/L)	2,412.9 (711.4)	0.112 (0.7590)	0.227 (0.5273)	−0.402 (0.2496)
Pharmacokinetic advantage	18.6 (6.8)	0.587 (0.0744)	0.716 (0.0198)	−0.334 (0.3453)

*BSA* body surface area, *BMI* body mass index, *AUC* area under the concentration-time curve, *β* estimated correlation coefficient

There were no statistically significant relationships between perfusate volume, BSA or BMI, and 24-hour glycemia or peak intraoperative blood glucose.

## Discussion

BSA is an imperfect but useful proxy to calculate drug doses, because of its association with circulating blood volume.^10^ Likewise, BSA has been used to estimate peritoneal volumes for peritoneal dialysis.^11^ BSA has been shown to be a predictor of outcomes following cardiopulmonary bypass, likely because of the association between low BSA and hemodilutional anemia in that setting.^12^ We hypothesized that the pharmacokinetics of HIPEC with oxaliplatin would be associated with BSA, due to its known association with circulating blood volume and peritoneal volume.

Our results suggest that in patients who receive a BSA-based oxaliplatin dose and carrier fluid volume titrated to achieve a desired flow rate, BSA is a predictor of systemic drug exposure and pharmacokinetic advantage. This is partially explained by the inverse relationship observed between perfusate volumes and systemic oxaliplatin levels, as perfusate volume was found to correlate with BSA. Patients with higher BSA had lower plasma oxaliplatin AUC over the 60-minute duration of HIPEC, and thus greater pharmacokinetic advantage, possibly because they also had larger circulating blood volumes with inadequate time for equilibration between the peritoneal and circulating blood compartments. Further studies should examine whether these relationships hold for patients who receive a set volume of carrier fluid, or a BSA-based volume of carrier fluid. We did not find that BMI was a significant predictor of pharmacokinetic parameters. The present study differed from a previous study showing such a relationship in terms of the patients’ diagnoses, the duration and technique of HIPEC, and surgical procedures and technique.^4^ We did not find obvious differences in pharmacokinetics on the basis of diagnosis, disease burden, or extent of peritonectomy, consistent with previous reports.^13^

We did not find statistically significant relationships between BSA or BMI and glycemia in our ten patients, but hyperglycemia was observed in all patients. Given the relatively small amount of oxaliplatin degradation in sodium chloride solution over the usual duration of HIPEC, use of normal saline in the perfusion circuit (after oxaliplatin reconstitution in 5 % dextrose), as has previously been described, may be considered.^3^,^6^

The present study shows that BSA can be used to predict the pharmacokinetics of HIPEC with oxaliplatin, likely due to the effects of circulating blood volume with inadequate time for drug equilibration. With the exception of metabolic derangements due to hyperglycemia, oxaliplatin HIPEC was well tolerated by all patients, suggesting that the range of systemic drug levels they experienced is safe. Patients with larger BSA, who had lower systemic drug levels, should therefore be able to tolerate higher total doses of oxaliplatin. This was a small cohort, however, and we did not prospectively analyze toxicity or efficacy, making it difficult to make clinical recommendations on the basis of our data alone. We therefore recommend further study of HIPEC dosing modified to achieve a desired intraperitoneal drug concentration for all patients, rather than a BSA-based total dose. For example, a system like ours, which titrates carrier fluid to achieve a minimum flow rate (which results in an variability in intraperitoneal drug concentrations) could be modified to use oxaliplatin at a set concentration, with the volume (and therefore the total dose) titrated to achieve the desired flow rate (which would result in equal intraperitoneal drug concentrations for all patients). Patients with larger BSA would then receive a higher total dose of drug, but, based on our data, the greater pharmacokinetic advantage in these patients would ensure that their systemic drug levels would remain tolerable. This method of dosing is more consistent with the observation that intraperitoneal oxaliplatin concentration, rather than total dose, is the chief determinant of HIPEC pharmacokinetics.^14^,^15^

The present study does not address the most important biodistribution endpoint, namely intratumoral drug concentrations, but instead uses peritoneal fluid concentration as a proxy. Few tissue analysis studies have been undertaken, and more are needed to optimize HIPEC administration and dosing in order to achieve the highest possible drug levels in tumor cells.^16^
